# Ungewöhnliche Iritis – Eine Fallvorstellung zur Listerienendophthalmitis

**DOI:** 10.1007/s00347-020-01252-w

**Published:** 2020-10-25

**Authors:** I. Bachmeier, M.-A. Gamulescu, H. Helbig, V. Radeck

**Affiliations:** Klinik und Poliklinik für Augenheilkunde, Universitätsklinikum Regensburg, Franz-Josef-Strauß-Allee 11, 93053 Regensburg, Deutschland

## Anamnese

Ein männlicher 53-jähriger Patient stellte sich mit seit 2 Wochen bestehender Rötung und Schmerzen am linken Auge in unserer Klinik vor. Seit dem Vortag bestand zudem eine ausgeprägte Visusminderung an dem betroffenen Auge. Bei Verdacht auf Konjunktivitis hatte eine Vorbehandlung mit antibiotika- und kortisonhaltigen Augentropfen über den niedergelassenen Augenarzt stattgefunden. Die Augenanamnese war ansonsten leer. Bekannte Vorerkrankungen aus dem rheumatischen Formenkreis oder chronisch entzündliche Darmerkrankungen (Morbus Crohn, Colitis ulcerosa) wurden verneint.

## Klinischer Befund

**Figure 1:**
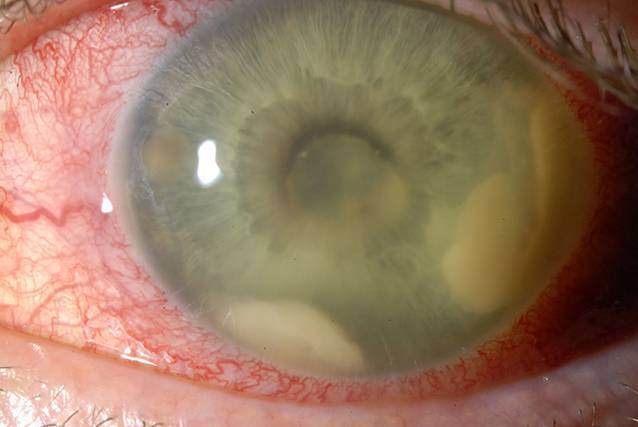

**Figure 2:**
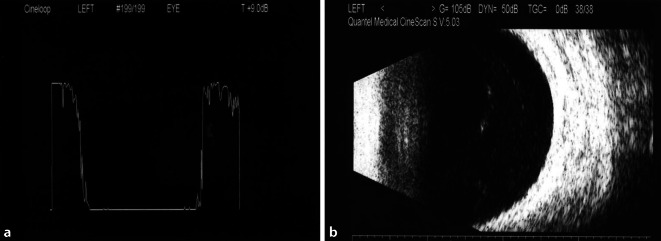

Der Visus des linken Auges lag bei Erstvorstellung bei Fingerzählen. Die Vorderkammer zeigte eine Fibrinreaktion, ein irregulär konfiguriertes helles Hypopyon und retrokorneale Beschläge in Form mehrerer kuppelförmiger Veränderungen aus festem weißlichem Material (Abb. 1). Die Hornhaut wies weder Infiltrate noch Epithelläsionen auf. Der Augeninnendruck betrug 18 mmHg. Fundoskopisch war aufgrund des massiven Vorderkammerreizes kein Einblick gegeben. Sonographisch ergab sich weder im A‑ noch im B‑Scan ein Hinweis auf eine Glaskörperinfiltration (Abb. 2).

Das Partnerauge wies einen vollen Visus (1,0 ohne Korrektion, Dezimalvisus) sowie reizfreie altersentsprechende Befunde des vorderen und hinteren Augenabschnitts auf.

## Verlauf und Therapie

**Figure 3:**
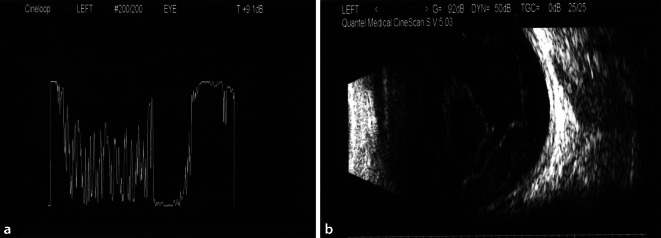

Der Patient wurde stationär aufgenommen, und es erfolgte zunächst bei Verdachtsdiagnose einer sterilen Uveitis anterior eine topische und später auch orale Therapie mit Kortikosteroiden. Bei nach 3 Tagen neu auftretenden Glaskörper-Spikes im Ultraschall-A-Scan und Trübungen im B‑Scan (s. Abb. 3) stellte sich der Verdacht auf ein infektiöses Geschehen mit Glaskörperinfiltration, sodass eine intravenöse Antibiose mit Ceftriaxon eingeleitet wurde. Zudem wurden intravitreale antibiotische (Amikacin, Vancomycin) und aufgrund einer Verschlechterung der Befunde später auch antimykotische (Amphotericin B) Injektionen eingegeben. Abgenommene Blutkulturen waren negativ, und auch in einer erneuten ausführlichen Allgemeinanamnese ließen sich keine klassischen Risikofaktoren für eine endogene Infektstreuung (i.v.-Drogenabusus, Immunsuppression, Venenkatheter o. Ä.) eruieren. Der Patient berichtete jedoch von Kopfschmerzen und grippeähnlichen Symptomen vor Beginn der Augensymptomatik und von seit vielen Jahren bestehenden rezidivierenden Analfisteln, welche bereits mehrfach operiert worden seien.

Im weiteren Verlauf zeigten sich das Fibrin und die Endothelbeschläge etwas abgegrenzter, es war jedoch weiterhin kein Funduseinblick möglich, und die Glaskörper-Spikes waren weiterhin stark ausgeprägt. Daher wurde, auch zum Keimnachweis, an Tag 9 eine Pars-plana-Vitrektomie (ppV) durchgeführt. Intraoperativ zeigte sich eine dichte Glaskörperinfiltration, die Netzhaut wies keine Infiltrate oder vaskulitischen Zeichen auf.

**Figure 4:**
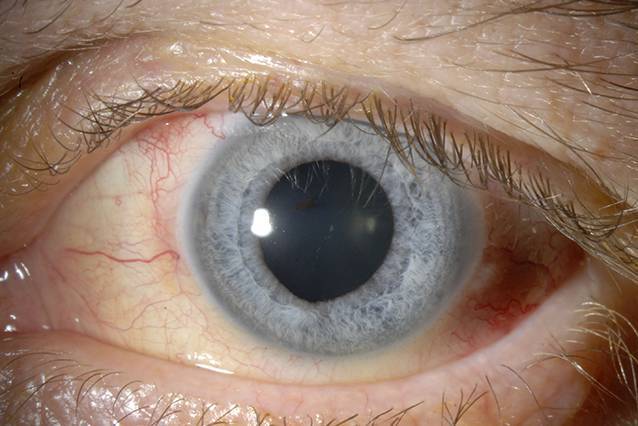

In den entnommenen Punktaten aus Vorderkammer und Glaskörper konnte schließlich *Listeria monocytogenes* als Erreger isoliert werden. Ebenso zeigte sich ein entnommener Analabstrich positiv für *Listeria monocytogenes*. Bei nun bekanntem Erreger wurde der Patient bezüglich einer möglichen Infektionsquelle befragt, wobei der Verzehr von Rohfleisch oder Rohmilch(‑Produkten) verneint wurde. Eine entsprechende mehrwöchige intravenöse antibiotische Therapie mit Ampicillin und Gentamicin wurde in unserer Klinik eingeleitet und heimatnah fortgeführt. Bei der Entlassung an Tag 17 war der Visus auf 0,2 ohne Korrektion angestiegen. Etwa 4 Wochen später bei der letzten Vorstellung lag der korrigierte Visus bei 0,3, und es zeigten sich bis auf vereinzelte Vorderkammerzellen reizfreie Befunde (Abb. 4).

## Diagnose

Endogene Endophthalmitis durch *Listeria monocytogenes*.

## Diskussion und Hintergrund

*Listeria monocytogenes* wurde in den 1920er-Jahren identifiziert und zu Ehren des im 19./20. Jahrhunderts lebenden britischen Mediziners und „Vater der antiseptischen Chirurgie“ Lord Joseph Lister benannt. Listerien sind grampositive, begeißelte, fakultativ anaerobe Stäbchenbakterien, die sich intrazellulär vermehren. Sie können direkt von einer Wirtszelle in die Nachbarzelle vordringen und somit anatomische Barrieren wie Schleimhäute, die Blut-Hirn-Schranke oder wie in diesem Fall die Blut-Retina-Schranke überwinden. Eine Ansteckung erfolgt in erster Linie über kontaminierte Lebensmittel, hier sind v. a. Rohmilch- und Rohfleischprodukte, Räucherfisch und Sprossen zu nennen [5]. Listerien sind fakultativ pathogene Erreger, sodass eine Infektion auch asymptomatisch verlaufen kann. Leichte Verläufe einer Listeriose können sich bei Immunkompetenten durch gastrointestinale Infekte oder wie im Falle unseres Patienten durch unspezifische, grippeähnliche Symptome äußern. Vor allem Immunsupprimierte, Ältere und Schwangere sind einem erhöhten Risiko ausgesetzt, komplizierte Verläufe einer Listeriose (Meningitis, Sepsis) zu erleiden oder zu versterben. Zudem kann eine Infektion von Mutter zu Kind transplazentar oder bei der Geburt erfolgen, was zum Abort oder zur Neugeborenensepsis oder -meningitis führen kann [5].

Eine okuläre Listeriose ist selten und äußert sich zumeist als exogen verursachte Konjunktivitis oder seltener auch Keratitis [7]. Deutlich seltener kommt es zur endogenen Listerienendophthalmitis, bei der der Erreger von einem meist ungeklärten Infektfokus über den Blutstrom unter Überwindung der Blut-Retina-Schranke in das Auge gelangt. Ihr Anteil an allen endogenen Endophthalmitiden wird mit 4 % angegeben [4], wobei endogene Endophthalmitiden wiederum nur 2–8 % aller Endophthalmitiden ausmachen [6]. Der Großteil der Endophthalmitiden tritt exogen verursacht nach Augenoperationen oder -traumata auf [4].

Seit 1967 wurden nur etwas mehr als 30 Fälle einer Listerienendophthalmitis publiziert; 27 dieser Fälle wurden in einem Review-Artikel von Bajor et al. hinsichtlich des Patientenkollektivs, des okulären Befunds, der Therapie und des Verlaufs analysiert [1]. Es existiert eine weitere Arbeit von Chersich et al., die zusätzlich unveröffentlichte Daten (nationale Gesundheitsbehörden, Kongressbeiträge etc.) einbezog und insgesamt 43 Fälle analysierte [2]. Die Patienten waren überwiegend männlich und im durchschnittlichen Alter von ca. 60 Jahren, ähnlich unserem Patienten. Etwa 50 % aller Patienten waren immunsupprimiert. Im Falle unseres Patienten lag keine eindeutige Immunsuppression vor, jedoch in Form der rezidivierenden Analfisteln eine chronische Erkrankung und potenziell erleichterte Eintrittspforte für Listerien in den Blutstrom. Unser Patient hatte wie die überwiegende Mehrzahl der Patienten über Schmerzen, Augenrötung und eine Visusminderung geklagt, etwa die Hälfte berichtete außerdem von grippeähnlichen Symptomen [1]. Typische Befunde sind ein massiver fibrinöser Vorderkammerreiz, Hornhautpräzipitate und ein Hypopyon, so auch bei unserem Patienten. Zur Infiltration des Glaskörpers kommt es meist erst verzögert. Die Tatsache, dass aufgrund dieser Befunde die Listerienendophthalmitis initial sehr häufig als sterile Uveitis anterior fehldiagnostiziert wird, erklärt den oft verzögerten Beginn einer effektiven Therapie. Zwischen Erstvorstellung und Einleiten der adäquaten Therapie lagen im Mittel 13 (4 bis 32) Tage [1] bzw. 8 (1 bis 38) Tage [2], im Falle unseres Patienten 10 Tage. Auch in unserem Fall hätte eine ppV zu einem früheren Zeitpunkt, z. B. bereits bei Verschlechterung unter der intravitrealen Antibiosegabe, erwogen werden können und dadurch aufgrund des früheren Keimnachweises rascher eine adäquate i.v.-Antibiose eingeleitet werden können. Bei fast allen untersuchten Patienten kam es zu einer deutlichen Augeninnendruckerhöhung, zudem wurde charakteristischerweise ein durch Irispigmentdispersion dunkles Hypopyon beschrieben [1], beides zeigte sich in unserem Fall nicht. Da sich die Listerienendophthalmitis insbesondere im anterioren Segment abspielt und die Netzhaut oft nicht betroffen ist, ist die Visusentwicklung bei adäquater und rascher Therapie meist erfreulicher als bei endogenen Endophthalmitisfällen im Allgemeinen, die in ca. 70 % in einem Visus von Fingerzählen oder schlechter resultieren [3]; 44 % [1] bzw. 60 % [2] der analysierten Listerienendophthalmitiden erreichten einen Endvisus von 0,1 oder besser, 26 % [1] bzw. 40 % [2] sogar einen Endvisus von mindestens 0,6. Auch unser Patient wies bei der letzten Vorstellung einen relativ guten Visus von 0,3 auf, der weitere Verlauf ist uns nicht bekannt, da keine Wiedervorstellung in unserer Klinik erfolgte.

## Fazit für die Praxis

- *Listeria monocytogenes* zählt zu den sehr seltenen Erregern einer endogenen Endophthalmitis.
- Typische Befunde sind ein dunkles Hypopyon, ein massiver fibrinöser Vorderkammerreiz und ein Tensioanstieg, weshalb es initial sehr häufig zur Fehldiagnose einer sterilen Uveitis anterior kommt.
- Klassischerweise beginnt die Infektion in der Vorderkammer mit einer sekundären Infiltration des Glaskörpers.
- Die Visusentwicklung ist bei adäquater und rascher Therapie meist vergleichsweise erfreulich.
